# PETCH-DB: a Portal for Exploring Tissue-specific and Complex disease-associated 5-Hydroxymethylcytosines

**DOI:** 10.1093/database/baad042

**Published:** 2023-06-10

**Authors:** Qinyun Cai, Zhou Zhang, Xiaolong Cui, Chang Zeng, Jiabin Cai, Jiajun Cai, Kai Wu, Xu Zhang, Yixiang Shi, Zoe Arvanitakis, Marc A Bissonnette, Brian C -H Chiu, Shi-Yuan Cheng, Chuan He, Wei Zhang

**Affiliations:** Department of Preventive Medicine, Northwestern University Feinberg School of Medicine, 680 N. Lake Shore Dr., Chicago, IL 60611, USA; Department of Preventive Medicine, Northwestern University Feinberg School of Medicine, 680 N. Lake Shore Dr., Chicago, IL 60611, USA; Department of Chemistry, The University of Chicago, 5735 S. Ellis Ave., Chicago, IL 60637, USA; Department of Preventive Medicine, Northwestern University Feinberg School of Medicine, 680 N. Lake Shore Dr., Chicago, IL 60611, USA; Zhongshan Hospital, Fudan University, 180 Fenglin Rd., Shanghai 200032, China; Huashan Hospital, Fudan University, 12 Wulumuqi Rd., Shanghai 200040, China; Department of Thoracic Surgery, The First Affiliated Hospital of Zhengzhou University, 1 Jianshedong Rd., Zhengzhou, Henan Province 450052, China; Department of Medicine, University of Illinois, 808 S. Wood St., Chicago, IL 60612, USA; Bionova (Shanghai) Medical Technology Co., Ltd., Building 42, 100 Banxia Rd., Shanghai 201318, China; Rush Alzheimer’s Disease Center, Rush University Medical Center, 1620 W. Harrison St., Chicago, IL 60612, USA; Department of Medicine, The University of Chicago, 5841 S. Maryland Ave., Chicago, IL 60637, USA; Department of Public Health Sciences, The University of Chicago, 5841 S. Maryland Ave., Chicago, IL 60637, USA; The Ken and Ruth Davee Department of Neurology, Northwestern University Feinberg School of Medicine, 303 E. Superior St., Chicago, IL 60611, USA; The Robert H. Lurie Comprehensive Cancer Center, Northwestern University Feinberg School of Medicine, 303 E. Superior St., Chicago, IL 60611, USA; Department of Chemistry, The University of Chicago, 5735 S. Ellis Ave., Chicago, IL 60637, USA; The Howard Hughes Medical Institute, The University of Chicago, 5735 S. Ellis Ave., Chicago, IL 60637, USA; Department of Preventive Medicine, Northwestern University Feinberg School of Medicine, 680 N. Lake Shore Dr., Chicago, IL 60611, USA; The Robert H. Lurie Comprehensive Cancer Center, Northwestern University Feinberg School of Medicine, 303 E. Superior St., Chicago, IL 60611, USA; Department of Chemistry, The University of Chicago, 5735 S. Ellis Ave., Chicago, IL 60637, USA

## Abstract

Epigenetic modifications play critical roles in gene regulation and disease pathobiology. Highly sensitive enabling technologies, including microarray- and sequencing-based approaches have allowed genome-wide profiling of cytosine modifications in DNAs in clinical samples to facilitate discovery of epigenetic biomarkers for disease diagnosis and prognosis. Historically, many previous studies, however, did not distinguish the most investigated 5-methylcytosines (5mC) from other modified cytosines, especially the biochemically stable 5-hydroxymethylcytosines (5hmC), which have been shown to have a distinct genomic distribution and regulatory role from 5mC. Notably, during the past several years, the 5hmC-Seal, a highly sensitive chemical labeling technique, has been demonstrated to be a powerful tool for genome-wide profiling of 5hmC in clinically feasible biospecimens (e.g. a few milliliter of plasma or serum). The 5hmC-Seal technique has been utilized by our team in biomarker discovery for human cancers and other complex diseases using circulating cell-free DNA (cfDNA), as well as the characterization of the first 5hmC Human Tissue Map. Convenient access to the accumulating 5hmC-Seal data will allow the research community to validate and re-use these results, potentially providing novel insights into epigenetic contribution to a range of human diseases. Here we introduce the PETCH-DB, an integrated database that was implemented to provide 5hmC-related results generated using the 5hmC-Seal technique. We aim the PETCH-DB to be a central portal, which will be available to the scientific community with regularly updated 5hmC data in clinical samples to reflect current advances in this field.

**Database URL** http://petch-db.org/

## Introduction

Epigenetic modifications play critical roles in gene regulation and disease pathobiology. DNA methylation, i.e. 5-methylcytosines (5mC) (i.e. methylation of the 5ʹ-cytosine at a CpG dinucleotide) are the most-investigated epigenetic modifications that have been implicated in various complex diseases. Biochemically, the 5mC can be oxidized into other modified cytosines. In particular, the 5mC in the human genome may be demethylated to produce 5-hydroxymethylcytsoines (5hmC) by the ten-eleven translocation (TET) family of methylcytosine dioxygenases in an active demethylation process (1). Of note, 5hmC modifications have been demonstrated to have a distinct genomic distribution from 5mC, showing enrichment within gene bodies and enhancer marks (2, 3). Functionally, in contrast to the well-established gene repression role of 5mC in promoters, 5hmC modifications have been associated with active gene expression in a tissue-specific manner (3). Of particular interest to translational research is the potential utility of 5hmC as a non-invasive epigenetic biomarker in circulating cell-free DNA (cfDNA) from liquid biopsies for the diagnosis and prognosis of complex diseases, such as early detection of human cancers (4) and complications from diabetes (5–8).

However, conventional epigenomic profiling methods, e.g. those bisulfite conversion-based technologies cannot distinguish the 5hmC from the more common 5mC modifications. To address this technical gap and accommodate the technical demand for robust profiling of genome-wide 5hmC in clinical biospecimens (e.g. cfDNA from plasma or serum) that often feature very low amount of DNA materials, several innovative technologies have been developed by the research community. Specifically, our team developed the 5hmC-Seal, an innovative technique for genome-wide 5hmC profiling that takes advantage of the highly sensitive and robust chemical labeling (9, 10). Combined with the next-generation sequencing (NGS) platforms, we have demonstrated the diagnostic and prognostic value of 5hmC in clinically feasible amount of cfDNA for several human cancers and diabetic complications (2, 5–7, 11–13). For instance, we developed a 32 gene-based diagnostic score that accurately distinguished early hepatocellular carcinoma (HCC) from non-HCC (validation set: area under curve = 88.4%), showing superior performance over α-fetoprotein (11). In another study, in patients with diffuse large B-cell lymphoma, a 29 gene-based prognostic score predicted event-free survival and overall survival more accurately than other established prognostic factors (13). In addition, we have generated the first 5hmC Human Tissue Map (i.e. bodymap) using the 5hmC-Seal and NGS in a variety of human tissue types and demonstrated tissue specificity of 5hmC (3), further supporting the potential clinical applications of 5hmC biomarkers, e.g. in the determination of tumor origin.

To provide convenient access to these 5hmC-related results to the research community and allow validation, re-use of these results, e.g. interpretation of (epi)genome-wide association studies [(E)GWAS)] and QTL mapping studies, we constructed the Portal for Exploring Tissue-specific and Complex disease-associated 5-Hydroxymethylcytosines (PETCH-DB), a database designed to be a portal for published results generated using the 5hmC-Seal technique. Specifically, the PETCH-DB provides internet-based searching and browsing functions for 5hmC features and modification levels, as well as relevant annotations (e.g. GeneCards) (14) and visualization (e.g. heatmaps, box plots and tables), in addition to downloadable raw results and supporting documents. The current PETCH-DB (v1.0) showcases the 5hmC Human Tissue Map (3) data and cfDNA-based biomarker discovery results from two case-control studies of liver cancer (11) and brain cancer (12). The PETCH-DB will be regularly updated to reflect current advances in this field.

## Methods

### Dataset description

**Figure 1.**
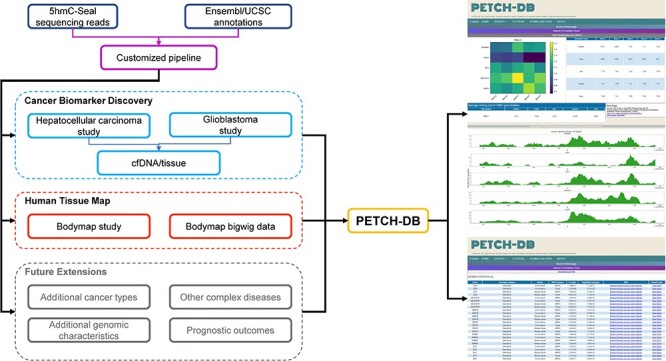
Overview of the database contents. The PETCH-DB (v1.0) aims to be a centralized portal for published 5hmC-Seal results processed by the previously published bioinformatic pipelines in primarily clinical samples. The figure shows two major data resources for Cancer Biomarker Discovery and Human Tissue Map (i.e. bodymap) and the Future Extensions as well.

Details about sample preparation, quality control, the 5hmC-Seal library construction and the NGS have been described in our previous publications (2, 9, 10). The raw 5hmC-Seal sequencing data included in the current version of PETCH-DB in the fastq format have been deposited into the NCBI Gene Omnibus (GEO) Database accompanying the respective peer-reviewed publication and are publicly available for downloading. All of the raw 5hmC-Seal sequencing data were processed using our published bioinformatic pipelines (2) and summarized for ∼20 000 gene bodies annotated by the GENCODE (15) for the human genome reference (hg19). In order to ensure a high-quality genomic coverage for the 5hmC data, the DESeq2 package (16) was used to normalize the raw count data, and the final list of gene bodies for each dataset was determined by customized threshold, considering specific sample size for each study (2, 11). Figure 1 shows the overview of the available datasets, bioinformatic processing and visualization features in the current PETCH-DB (v1.0).

### Tissue-specific 5hmC

To characterize tissue-specific 5hmC modifications, we obtained the genome-wide 5hmC data from a Human Tissue Map of 5hmC (GSE144530) published by our team through a collaboration with Bluestar Genomics Inc. (3), in which 19 fresh-frozen tissue specimens, corresponding to 10 organ systems, were collected from five non-cancer donors through patient surgery or postmortem provided by ProteoGenex Inc. in California. Donors were 50–70 years of age and balanced between males and females for all tissue samples. Either donors or next of kin were consented for DNA, RNA and protein analysis through an ethics board approved study protocol at ProteoGenex. The genomic DNA samples isolated from these tissues were subjected to the 5hmC-Seal profiling. In total, the current PETCH-DB covers 96 samples with available 5hmC data that were processed, summarized and normalized as described in the accompanying peer-reviewed publication (3).

### Cancer-associated 5hmC

The current PETCH-DB showcases differential analysis results obtained from the 5hmC-Seal profiles in circulating cfDNA from two case-control studies in China: HCC (11) and glioblastoma (GBM) (12). Specifically, for the liver cancer dataset, we processed the raw 5hmC-Seal data for ∼1200 patients with HCC and ∼900 healthy controls (GSE112679); and for the GBM dataset, ∼110 patients with GBM and ∼110 healthy controls (GSE132118) were processed and included in the current PETCH-DB. The normalized 5hmC levels were compared between cases and controls, controlling for age and sex, using DESeq2 (16). The PETCH-DB serves key results from the differential analysis, including fold changes, *P*-values and annotation information for each tested 5hmC feature in the HCC or GBM study.

## Backend service implementation

### Database architecture and programming

**Figure 2.**
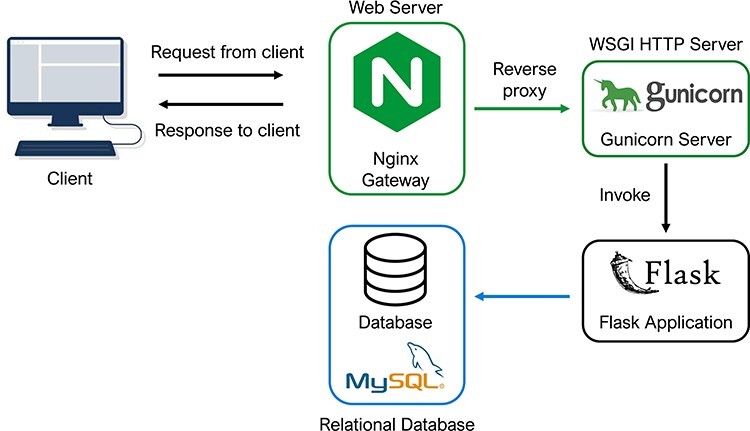
Database architecture and programming. The architectural flow of the PETCH-DB is shown. NGINX and Gunicorn are used to provide a server gateway interface to the flask application, which transmits requests to the MySQL database, thus offering a flexible framework for future expansion of the database.

Technically, the PETCH-DB was built on an extensible and modular architecture, with the intention of this flexible database schema to allow the incorporation of new data from other sources, for example, when we expect to update the database to serve new studies. The architecture of the PETCH-DB is shown in Figure 2. Briefly, the PETCH-DB was implemented based on Flask (v2.0.2), a web development framework in Python. This is a cloud-based database hosted by Amazon Elastic Compute Cloud, a resizable and efficient cloud computing service. The application runs on a high-performance HTTP server, NGINX (v1.18.0), with Gunicorn as the web server gateway interface. All of the data (e.g. normalized 5hmC levels and differential results between cases and controls) are stored in Amazon Simple Storage Service and Amazon Relational Database Service for MySQL (v8.0).

### User interfaces

**Figure 3.**
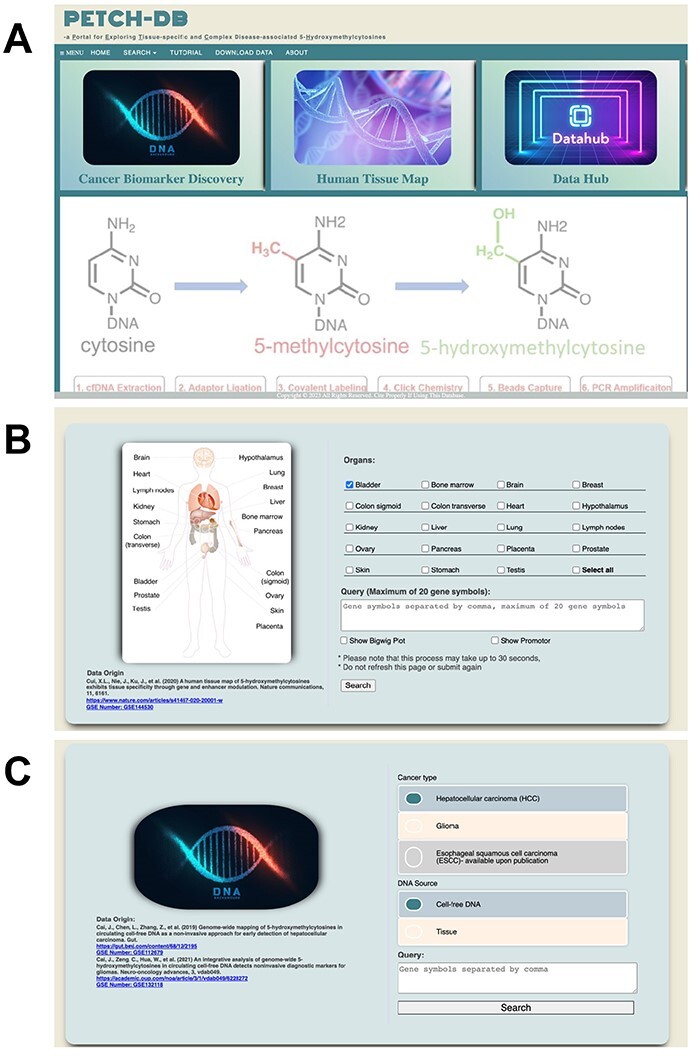
User interfaces. Screenshots are showcased for (**A**) the main interface, (**B**) Human Tissue Map and (**C**) Cancer Biomarker Discovery.

The PECTCH-DB has an accessible user interface developed using HTML, CSS, jQuery and JavaScript. The user interfaces for the PETCH-DB currently supports the interrogation of ‘Cancer Biomarker Discovery’ and ‘Human Tissue Map’, which represents the main entry point for the case-control studies and the 5hmC tissue map, respectively (Figure 3A). Additionally, there is also an interface—‘Data Hub’ for downloading the served 5hmC results in the current version. From the home page, users can also select the entry point from a menu, which also provides access to a brief tutorial and contact information for questions. Once the entry point of interest is selected, users will be presented with an interface to perform queries from the respective resource. The PETCH-DB supports official gene symbols and genomic regions (hg19). If users search the database with multiple genes but containing a gene symbol that does not exist, the database will only return results for valid gene symbols. To help users accurately find gene symbols, the PETCH-DB uses a jQuery script to provide auto-completion with gene locus location attached.

A brief introduction of the user interfaces for the two current major result resources is as follows:

Cancer Biomarker Discovery—At the main query interface of the case-control studies (Figure 3C), users can select the specific peer-reviewed study, currently HCC or GBM, and enter gene symbols or genomic regions (hg19) of interest. Although the majority of samples included in these studies are cfDNA based, sometimes tissue samples were also included for comparison or technical confirmation in these studies. Users therefore are required to specify the DNA source at this interface, with cfDNA being the default. Users who are interested in individual-level sample information, such as tumor stage, age and gender will have to contact the corresponding authors of respective publications, because these variables are not primary targets of the PECTCH-DB.

Human Tissue Map—At the main query interface of the 5hmC tissue map resource (Figure 3B), users can select the tissues and organs of interest (at least one tissue type), and enter the gene symbols (i.e. official gene symbol, single or multiple or genomic region based on hg19) in the query box. Users can choose to show gene bodies (default) and promoters (optional), as well as the BigWig plots for the Genome Browser view of the data.

### Outputs of the database

After valid queries are processed, the PETCH-DB will display customized outputs for the respective resources.

Human Tissue Map—The PETCH-DB provides data visualization on the specified genes (i.e. gene bodies as default with promoter as optional) or genomic regions with a heatmap and genome peak track across the selected tissue types. If a single gene is queried, a heatmap will show each donor sample available for that gene. When multiple genes are queried, the average 5hmC values across the five donor samples will be used to represent each gene in the resulting heatmap. Since the primary purpose of the heatmap is to provide the visualization of 5hmC distribution across different tissue types, no clustering is performed on the data. Instead, the normalized 5hmC data are provided in a table and made available for downloading for users’ convenience, e.g. additional analysis or visualization. If the ‘BigWig plot’ option is selected, the database will also output a view of the distribution of 5hmC data (i.e. peak tracks) across the selected genic regions for each selected tissue type.

Cancer Biomarker Discovery—For users who are interested in the results from the case-control cancer biomarker discovery studies, the PETCH-DB will provide key results from differential analysis (e.g. *P*-values, fold changes) in a resulting table and visualization of the differential analysis between cases and controls for a selected study using box plots. The search results are linked with the GeneCards (14) for each gene using official gene symbols (hg19) to provide more information about the search target.

## Results

**Figure 4.**
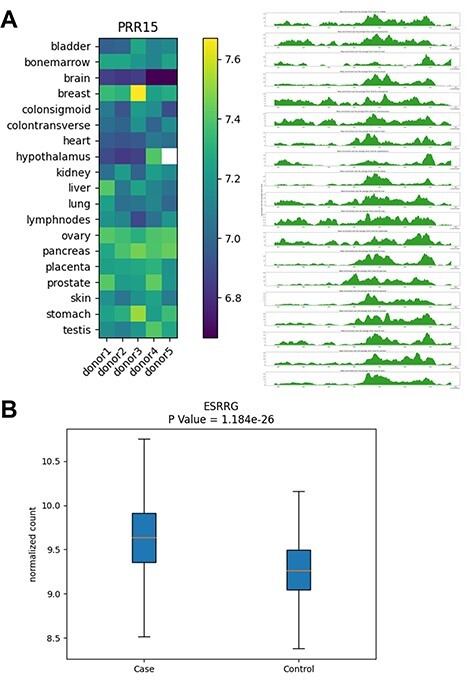
Examples of database outputs. Examples of database outputs are showcased for (**A**) Human Tissue Map: the PRR15 gene shows brain-specific 5hmC modification, compared with various tissue types and (**B**) Cancer Biomarker Discovery: the ESRRG gene shows higher 5hmC level in liver cancer patients relative to controls from a liver cancer study.

We envision that the PETCH-DB, through providing convenient access to the results from studies using the 5hmC-Sea technique, will be a central portal that can benefit the research community in several ways. For example, this database can be used to support such applications as improving data interpretation of GWAS or EWAS findings, prioritizing results from other association studies for further functional studies (e.g. by providing more evidence for the association of 5hmC with a disease or tissue type) and linking 5hmC associated with a disease in cfDNA to other multi-omics data (e.g. RNA-seq) generated in clinical samples. Figure 4 shows two examples, with the aim to demonstrate the main outputs from the PETCH-DB.

### Application example 1—tissue-specific modification pattern of PRR15

Our previous published research in brain cancer (12) identified the 5hmC modification level of the PRR15 gene (encoding ‘proline rich 15’), specifically its gene body modification as one of the cfDNA-based diagnostic markers for gliomas. By searching for this gene in the Human Tissue Map provided by the PETCH-DB, we found that the PRR15 gene had the lowest 5hmC modification levels in the brain from all five donors compared with other organs (Figure 4A), thus providing the information about the baseline tissue-specificity of PRR15 gene and its potential relevance to the brain biology, although this gene was initially identified only in plasma cfDNA derived from patients with brain cancer.

### Application example 2—association of ESRRG gene with liver cancer

The ESRRG gene (encoding estrogen related receptor gamma) was identified as a 5hmC marker component of a cfDNA-based diagnostic model for early HCC, according to our previously published research on the 5hmC biomarker discovery in liver cancer (11). By searching for this gene in our PETCH-DB database’s Cancer Biomarker Discovery, we confirmed that the ESRRG gene had higher 5hmC levels in HCC patients (i.e. cases), relative to the controls in an automatically generated box plot (Figure 4B). The query results also link to the reported *P*-value, fold change as well as the GeneCards (17) annotation, thus offering a convenient access to the basic findings from the HCC study, which otherwise could be time-consuming to locate from the original publication.

## Conclusion and future development

We developed a database with convenient user interfaces to provide centralized and convenient access to a range of results from our previous 5hmC-related studies, currently the Human Tissue Map and two clinical biomarker discovery studies of liver cancer and GBM. The PETCH-DB was designed to provide an authoritative presentation of the results generated from one of the leading groups of novel biomarker discovery and basic research using the 5hmC-Seal technique in liquid biopsy or tissue samples. Of note, our team will regularly update the PETCH-DB to include accumulating data from our on-going and previously published research projects, such as biomarker discovery for early detection of other cancers (e.g. colorectal cancer, pancreatic cancer, lung cancer and esophageal cancer) and complex diseases [e.g. diabetic vascular complications (5–7), Alzheimer’s disease (18) and aging (8)] as well as other relevant research [e.g. subtyping (19), prognosis (13) and race/ethnicity disparities (20)], after the primary results from these studies are published in peer-reviewed journals and the raw sequencing data have been deposited into the NCBI GEO Database. To our knowledge, our team is the largest group that has contributed to the 5hmC-Seal data generated using this technique in clinical samples. We intend to incorporate future extensions into subsequent versions of the PETCH-DB database. Although issues like technical batch effects and sample authentication could be challenging, these extensions could include, for example, additional cancer types, more complex diseases such as diabetic complications and cardiovascular diseases (21), additional genomic characteristics such as promoter regions and lung non-coding RNAs (22) and other clinical outcomes such as prognosis. In summary, we envision this new database of 5hmc-related studies will be a unique resource to support the research community’s effort of enhancing our understanding of the roles of these under-investigated epigenetic modifications in human diseases, as well as to facilitate the development of powerful epigenetic tools for precision medicine.

## Data Availability

The data underlying this article are available in the NCBI Gene Expression Omnibus at https://www.ncbi.nlm.nih.gov/geo/, and can be accessed with accession numbers: GSE112679, GSE132118, and GSE144530.
